# Adapting and usability testing of the Kansas city cardiomyopathy questionnaire (KCCQ) in a heart failure clinic in Tanzania: the Swahili KCCQ

**DOI:** 10.1186/s12872-023-03265-0

**Published:** 2023-05-06

**Authors:** Pilly Chillo, Jackson Mlay, Precious W Akanyirige, Naizihijwa Majani, Mohamed Janabi, Sylvia Kaaya, Claudia Hawkins, Lisa R Hirschhorn

**Affiliations:** 1 Department of Internal Medicine, Muhimbili University of Health and Allied Sciences, Dar es Salaam, Tanzania; 2 Jakaya Kikwete Cardiac Institute, Dar es Salaam, Tanzania; 3 Feinberg School of Medicine, Northwestern University, Evanston, USA; 4 Department of Psychiatry and Mental Health, Muhimbili University of Health and Allied Sciences, Dar es Salaam, Tanzania

**Keywords:** Patient-reported outcomes, Quality of life, Patient-centered care, Heart failure, Sub-Saharan Africa, Tanzania, KCCQ, Swahili

## Abstract

**Background:**

The integration of patient–reported outcome measures (PROMS) into health care delivery systems is being increasingly recognized as an important component of quality, person-centered care, especially for chronic illnesses like congestive heart failure (CHF). However, while PROMS are increasingly being used to follow up CHF patients in high income countries, their use in sub-Saharan Africa is still limited. We adapted the Kansas City Cardiomyopathy Questionnaire (KCCQ-23), an internationally validated, CHF-specific PROM and tested its use in measuring outcomes in an outpatient CHF clinic at a cardiac referral hospital in Tanzania.

**Methods:**

Adaptation of the KCCQ-23 included translation into Swahili by linguistic experts, in-depth cognitive debriefing in native Swahili–speaking CHF patients, and input from Tanzanian Cardiologists, PROMS experts, and the tool developer. Using a cross-sectional design, we tested the usability and observed the results of the translated KCCQ-23 in a convenience sample of 60 CHF patients attending outpatient clinic at the Jakaya Kikwete Cardiac Institute (JKCI) in Dar es Salaam.

**Results:**

The survey was successfully completed by 59 (98.3%) of 60 enrolled participants. The mean (SD) age of participants was 54.9 (14.8) years (range 22–83), 30.5% were women and 72.2% had class 3 or 4 New York Heart Association (NYHA) symptoms at enrollment. The overall KCCQ-23 score was low, with a mean (SD) score of 21.7 (20.4) indicating generally very poor to poor patient reported outcomes in this population. The mean (SD) scores for the specific KCCQ-23 domains were 15.25 (24.2) for social limitation, 23.8 (27.4) for physical limitation, 27.1 (24.1) for quality of life and 40.7 (17.0) for self-efficacy. No socio-demographic or clinical characteristics were associated with their overall KCCQ-23 scores. Comparing the short version (KCCQ-12) with the full KCCQ-23 revealed excellent correlation between the two (*r* = 0.95; *p* < 0.0001).

**Conclusions:**

We successfully translated a validated tool, the Swahili KCCQ, for use in improving the care of patients with CHF in Tanzania and a broader population of Swahili–speaking patients. Both the Swahili KCCQ-12 and KCCQ-23 can be used, with similar outcomes. Work to expand the use of the tool in the clinic and other settings is planned.

**Supplementary Information:**

The online version contains supplementary material available at 10.1186/s12872-023-03265-0.

## Background

The global burden of congestive heart failure (CHF) is expected to increase as a result of several factors such as an aging population, improved outcomes of acute cardiovascular events, and a rise in cardiovascular risk factors globally [1, 2]. Most of the projected CHF increase is expected to occur in low- and middle-income countries (LMICs), including across countries in sub-Saharan Africa (SSA). This projection identifies a need to strengthen care for this population by ensuring the provision of the person-centered, longitudinal care needed to improve treatment outcomes in CHF [1, 3].

Traditionally, measures of patients’ progress in CHF have been based on providers’ assessment of clinical status and laboratory or other diagnostic tests. While these are essential to help achieve some of the goals of quality, person-centered care among CHF patients, there is also a growing recognition of the importance of assessing patients’ clinical status through patient-reported outcome measures (PROMS) [4, 5]. PROMS are standardized tools on a patient’s health condition and health behaviors which are reported directly by the patient without interpretation by anyone [6]. PROMS therefore give the patients and their caregivers a voice in their care. When used systematically, PROMS yield quantitative data that enables comparison between individual patients, patient groups, and providers across different settings as well as contribute information for potential improvement in the quality of care at a health facility [6].

The Kansas City Cardiomyopathy Questionnaire (KCCQ) is a 23-item, self-administered PROM developed to independently measure a patient’s perception of their health status, which includes CHF symptoms, impact on physical and social function, and how CHF impacts their quality of life within a 2-week recall period [7]. The tool has been extensively validated and is one of the most commonly used tools by providers during follow-up of persons with CHF [8]. The tool measures patient–reported outcomes (PROs) with summary scores as well as individual domain scores in Physical Limitation, Total Symptoms, Quality of Life and Social Limitation [8]. In addition, studies have found that a subset of the measures, the KCCQ-12 scale, is a valid and reliable PROM which is highly correlated with the full form, offering a shorter tool for clinical use [9]. The use of PROMS like the KCCQ has been increasing both in clinical trials [10–13], and in general care [14–16] in a growing number of high income countries (HICs). Additionally, several groups, including the World Health Organization (WHO) and the Organization for Economic Cooperation and Development (OECD), are calling for integration of PROMS into strengthened health care delivery systems for all chronic diseases across all countries [17].

The use of PROMS in African countries is steadily growing in HIV care [18, 19], but only a few studies have reported on use of PROMS in CHF care in the region [20, 21]. In Uganda, Okello et al. validated the KCCQ-23 in patients admitted with acute CHF in Mbarara and found the KCCQ-23 to have high internal consistency, with lower KCCQ-23 scores predicting mortality at 3 months [20]. In more recently published data from the Global Congestive Heart Failure study, which included African countries, the KCCQ-12 score was found to be a strong, independent predictor of all-cause death and CHF hospitalization in mildly and severely symptomatic CHF patients [21]. In this study, patients from Africa had the lowest scores of any group (39.5 ± 0.3), with the highest scores reported by patients from HICs in Western Europe (62.5 ± 0.4); these findings reemphasize the high disease burden in patients from African countries [21]. However, the KCCQ had not been translated into Swahili. With over 108 million speakers, Swahili is the most widely spoken language in Africa [22], and therefore the use of the KCCQ is still limited among native Swahili speakers.

In this study we report on the process we undertook to translate and adapt the KCCQ-23 into Swahili and the results of a pilot study using this Swahili KCCQ-23 to assess PROs in CHF patients in an outpatient cardiology clinic in Tanzania. The results contribute to work to increase patient–centered outcome research, inform planning of clinical interventions to improve patient outcomes including in symptoms, functioning and quality of life, and empower CHF patients in Tanzania and the Eastern, Central, and Southern African regions where Swahili is widely spoken [23].

## Methods

### Study design, site, population and duration

We conducted a cross–sectional study in the outpatient clinic of the Jakaya Kikwete Cardiac Institute (JKCI) in Dar es Salaam to understand the usability of the tool and to report on PROs resulting from the KCCQ-23. The study was part of a larger effort to explore feasibility, adoption and appropriateness of measuring PROMS and patient-reported experiences among CHF patients in a referral clinic in Tanzania. JKCI is the major referral cardiac hospital in Tanzania, receiving patients from all over the country. The outpatient clinic is run from Monday to Friday and attends to an average 200 patients per day, of whom 30% have CHF [24]. On average, there are seven doctors (general physicians and specialist cardiologists) attending the clinic at any time, with an average doctor:patient ratio of 1:30 per clinic. The study took place between March 2020 and March 2021, delayed in part due to COVID-19 restrictions.

### Selection of PROMS

We reviewed both disease-specific (CHF-specific symptoms) and general PROMs (quality of life, self-efficacy, communication, health beliefs) which had been used in Swahili, and those previously used in SSA. Following discussions with experts in the field of PROMS, as well as with cardiologists at JKCI and at Northwestern University (United States), the KCCQ-23 was selected as the most appropriate tool to be used based on length, use in other countries in the region, and validation in clinical care. We chose to start with the KCCQ-23 tool, with a secondary goal of comparing KCCQ-23 and the shorter KCCQ-12. The KCCQ-23 and KCCQ–12 scores range from 0 to 100 and are classified as very poor to poor (0 to 24), poor to fair (25 to 49), fair to good (50 to 74) and good to excellent (75–100).

### Translation, cognitive debriefing and adaptation of the KCCQ-23

The translation, cognitive debriefing and adaptation of the KCCQ-23 followed established procedures already validated and widely used by the developer of the tool [25]. The process was carried out by MAPI/ICON Language Services Company which has deep experience in the linguistic validation process for the KCCQ-23 into different languages [26]. The MAPI/ICON translation followed the ISPOR (International Society for Pharmacoeconomics and Outcomes Research)-guided process as recommended by the US Food and Drug Administration (FDA) and EMA (European Medicines Agency) regulatory authorities for Patient–Reported Outcome Assessments tools [27]. The English back translation was then compared with the original KCCQ-23 by experts in PROMS and CHF care (led by LH and PC, respectively). The translated KCCQ-23 then underwent rigorous cognitive debriefing in a convenience sample of five CHF patients who were native Swahili speakers to elicit feedback on relevance, comprehensibility, language, ambiguity, and any misunderstanding of the concepts by the patients. A detailed process of each step is shown in Table 1. The final tool (Appendix 1) was approved and agreed upon by experts in CHF who are both fluent in English and native Swahili speakers (PC, JM, NM, MJ).

**Table 1:** The KCCQ-23 translation process into Swahili

Step	Activity	Outcome
1.	Conceptual analysis of the concepts investigated in the KCCQ-23 translated by MAPI Language Services’ project manager and validated by the Author (Prof. Spertus).	Establishment of version 1 of the Swahili KCCQ-23
2.	Dual forward translations conducted by two native Swahili speaking linguists.	
3.	Reconciliation of forward translations by an additional independent native Swahili speaking linguist residing in Tanzania.	
4.	Report summarizing the issues encountered and the solutions suggested.	
5.	Quality control by MAPI.	
6.	Single back translation of reconciled forward translation done by a new native Swahili linguist who has never seen the source material.	Blinded back translation
7.	Reconciliation and analysis of back translation against the original questionnaire by the in-country Swahili linguist and by MAPI.	Establishment of version 2 of the Swahili KCCQ-23
8.	Report by the in-country Swahili linguist summarizing the issues encountered and solutions suggested.	
9.	Quality control by MAPI, and discussion with the Swahili linguist.	
10.	Review by a clinician native Swahili speaker, residing in Tanzania of the translation version 2, in order to get input from a medical expert in cardiology on the translation as to the domain-specific terminology.	Establishment of version 3 of the Swahili KCCQ-23
11.	Suggestions made by clinician on the translation checked against the conceptual information obtained by from the Author.	
12.	Analysis and quality control of feedback by MAPI and the in-country Swahili linguist.	
13.	Clinician recruitment of 5 patients with heart failure who are Swahili native speakers residing in Tanzania.	Cognitive debriefing and adaptation of the Swahili KCCQ-23
14.	In-depth individual interviews conducted by the in-country Swahili linguist investigating the clarity, understandability and acceptability of the translation version 3. The participants commented on their understanding of each item and suggested alternative formulations in the case of problematic wording.	
15.	Analysis and report by the in-country Swahili linguist summarizing the issues encountered and solutions suggested.	Establishment of versions 4 of the Swahili KCCQ-23
16.	Quality control by MAPI and discussion with the Swahili linguist based on the results of the clinician’s and respondents’ feedback,	
17.	Dual proofreading of the language version: one conducted by an independent translator and one by in-country Swahili linguist.	Final version of the Swahili KCCQ-23 tool
18.	Analysis and report summarizing the issues encountered and solutions suggested.	
19.	Quality control by MAPI.	

### Testing the usability and performance of the Swahili KCCQ-23 in a cardiac outpatient clinic at JKCI

Using a cross-sectional study design, the translated and adapted KCCQ-23 was administered to a convenience sample of 60 adult CHF patients being seen in the clinic for a previously scheduled appointment. The survey also captured additional socio-demographic and clinical data including age, sex, education, residence, type of visit, presence of comorbidities, and common symptoms of HF. Patients were eligible if they were aged ≥ 18 years and known to have a diagnosis of CHF (from any cause) of at least six months duration based on physician diagnosis. Briefly, in the morning of each cardiac outpatient clinic day, a trained research assistant would identify possible participants and determine their eligibility. Once eligible, the patient and/or their caregiver would be approached for consent. Once consented, the patient and/or caregiver was given the paper questionnaire to fill in their responses. The research assistant then collected the questionnaires which were later entered into a REDCap (Research Electronic Data Capture) database created for this study.

### Data analysis

Descriptive statistics were used to describe the range of scores, including measures of central tendency (mean, median), spread (standard deviation, range) and response category frequencies. Because the results were not normally distributed, we used the Mann-Whitney U test to explore differences in the KCCQ-23 score and selected dichotomous variables (sex, education secondary or higher, NYHA 3 or 4) and linear regression for continuous variables (age). We also calculated correlation between the KCCQ-23 and KCCQ-12 summary scores using Pearson Correlation Coefficient to explore the potential for using the shorter version in clinical practice. All analyses were done using STATA 17.0 (College Station, Texas).

## Results

### Socio-demographics/Patient characteristics

Overall, 59 (98.3%) of the 60 enrolled individuals were able to complete and return the KCCQ-23 tool. One patient did not return their questionnaire to the research assistant and was therefore excluded in the analysis. One–third (30.5%) of the 59 respondents were female, with 67.5% of respondents aged ≥ 50 years, and 32.2% having completed secondary school or higher (Table 2). Most respondents were from the Eastern region of Tanzania, where JKCI is located. The majority were being seen for a follow-up (54.2%). The population had significant symptoms of CHF with close to three-quarters (72.2%) with New York Heart Association (NYHA) Classification of stage 3 or 4, and most reporting at least one current symptom of dyspnea, angina, orthopnea or edema.

**Table 2:** Socio-demographic characteristics of the studied population

Socio-demographics characteristics	Number (%) Total 59
Age groups (years)	
<40	10 (16.95)
40–49	10 (16.95)
50–59	15 (25.42)
60–69	13 (22.03)
≥70	11 (18.64)
Female	18 (30.5)
Highest education level completed	
Less than primary	15 (25.42)
Completed primary	24 (40.68)
Completed Secondary	13 (22.03)
University or higher	6 (10.17)
Missing	1 (1.69)
Live in Eastern Region	36 (61.02)
Home–Owner	49 (83.05)
Employed	26 (44.07)
Visit Type	
Follow–Up Visit	32 (54.24)
First visit	27 (45.76)
Insured	31 (52.54)
NYHA Class	
I	4 (6.78)
II	12 (20.34)
III	40 (68.80)
IV	2 (3.39)
Missing	1 (1.69)
Self–Reported Symptoms	
Dyspnea	46 (79.31)
Orthopnea	38 (64.41)
Edema	44 (74.58)
Angina	41 (69.49)
Self–Reported Comorbidities	
Hypertension	34 (57.63)
Diabetes	5 (8.47)
Kidney Disease	4 (6.78)
>1 comorbidity	39 (66.10)

### KCCQ-23 results

**Fig. 1:**
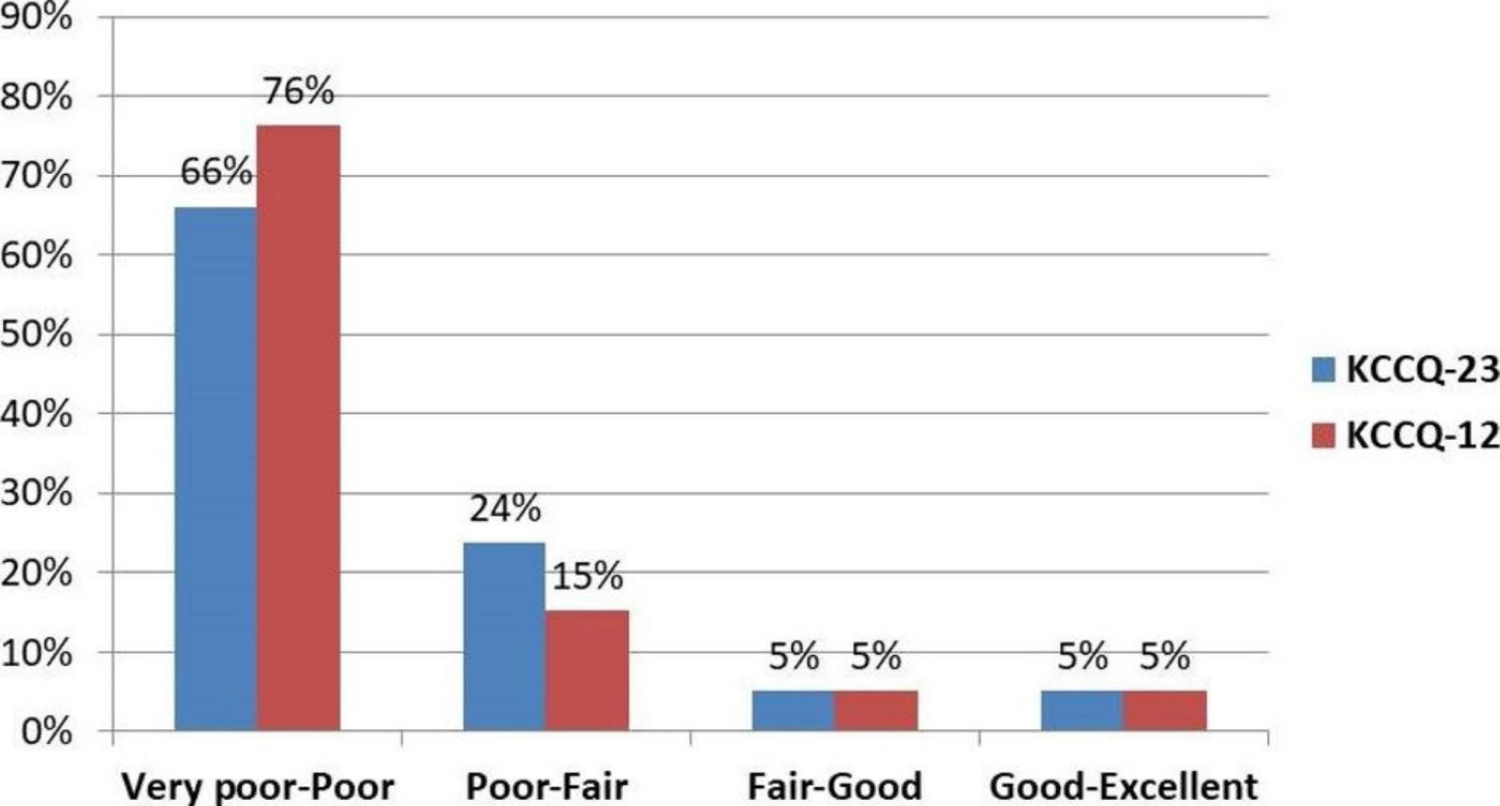
Percent Distribution of KCCQ-23 and KCCQ-12 Categories Among Heart Failure Patients

The scores for the KCCQ-23 were low, with a mean of 21.7 (SD 20.4) and median of 17.5 (IQR 4.2, 32.8) (Table 3). Only 5.08% scored in the ‘Fair to Good’ (> 49 to 74) and 5.08% in the ‘Good to Excellent’ (> 74–100) categories (Fig. 1). Scores were also low across the specific domains, with the lowest in Social Limitation (mean 15.25, SD 24.2) and highest in Self–Efficacy (mean 40.7, SD 17.0). Physical Limitations were particularly prominent in those requiring more efforts such as climbing stairs or hurrying (72.9% extremely limited) and two-thirds reporting severe limitations in many recreational or social activities such as visiting families, relationships and household chores. Over three quarters reported fatigue and dyspnea three or more times per week, 57.6% had extremely limited enjoyment of life and 45.8% were not at all satisfied with their current quality of life living with CHF. The majority of respondents felt somewhat (62.7%) or mostly (6.8%) confident in their ability to manage their CHF and prevent it from worsening. No patient or visit factors were found to be associated with overall KCCQ-23 score (Appendix 2). Comparing the KCCQ-23 and shorter KCCQ-12 scores found high correlation, with Pearson correlation coefficient of 0.95, p < 0.0001.

**Table 3:** Mean and Median Calculated Scores for KCCQ-23 and KCCQ-12: Overall (range 0-100) and Sub–Scores

	KCCQ-23 Mean (SD)	KCCQ-23 Median (IQR)	KCCQ12 Mean (SD)	KCCQ12 Median (IQR)
**Overall Score** ^**1**^	21.7 (20.4)	17.5 (8.3, 26.6)	20.8 (19.6)	16.2 (6.3, 24.5)
**Physical Limitations**	23.8 (27.4)	12.5 (0, 33.3)	24.3 (28.5)	8.3 (0, 33.3)
**Total Symptom Score** ^**2**^	20.7 (22.0)	12.5 (3.6, 28.6)	-	-
Symptom Stability	16.9 (22.0)	0 (0, 25.0)	-	-
Symptom Frequency	21.4 (21.8)	14.6 (6.3, 29.0)	21.4 (21.9)	14.6 (6.3, 29.2)
Symptom Burden	20.1 (23.8)	8.3 (0, 25.0)	-	-
**Self-Efficacy**	40.7 (17.0)	50.0 (25.0, 50.0)	-	-
**Quality of Life**	27.1 (24.1)	25.0 (8.3, 41.7)	22.7 (23.7)	25.0 (0, 37.5)
**Social Limitations**	15.3 (24.2)	0 (0, 25.0)	14.8 (24.1)	0 (0, 25.0)
**Clinical Summary Score** ^3^	22.3 (21.7)	18.8 (4.2, 32.8)	**-**	

SD: standard deviation, IQR interquartile range

^1^ Calculated from the mean of the Total Symptom Score, Physical Limitation, Quality of Life Score, Social Limitation Score; range 0-100

^2^ Calculated from the mean of Symptom Frequency and Symptom Burden Scores; range 0-100

^3^ Calculated from the mean of the Physical Limitation Score and Total Symptom Score; range 0-100

## Discussion

We report on the successful linguistic and cultural translation of the KCCQ-23 into Swahili, and a preliminary assessment of its usability in a cardiac outpatient clinic in Tanzania. We found in pilot testing that the tool was able to be self-administered and identified significant levels of patient-reported disability related to their CHF. In addition, high correlation as seen between the full tool and the shorter version, offering the potential for use of a simpler tool in studies and clinical care.

Although previous studies have reported use of the KCCQ-23 in Africa and among native Swahili speakers [20, 21], ours is the first to formally translate and follow the guideline-recommended methods for tool translation and adaptation [27]; our form is officially the approved Swahili KCCQ-23 by the developer. Marsh et al. observed in South Africa that while not always done, adherence to standards for tool translation is important [28] to fully obtain the intended outcomes [27] and minimize bias [21]. The rigorous process for translation into Swahili, the most widely spoken language in Africa [22], provides the opportunity to understand use of this standard tool in a large Africa population, therefore increasing the voice of patients with chronic CHF across many regions. While still not widely in use, recent reviews have highlighted the importance and growing use of PROMS in cardiovascular disease in Africa as the burden of these chronic diseases increase along with the availability of care [23]. Other studies have also supported the use of PROs including work associating levels of stress with coronary artery disease in Turkey [29]. A recent set of recommendations for outcomes among patients with hypertension in LMICs included some PROMS, including quality of life and erectile dysfunction in addition to more traditional clinical outcomes and patient experiential quality [30].

We used a self-administered, paper-based KCCQ-23. Although there was no systematic assessment of the time used by the patients to fill in the questionnaire, all patients were able to fill the questionnaire during their waiting time at the clinic. Furthermore, most patients could fill in the questionnaire without asking for clarification or for help, indicating the tool was easily understood by the patients. These results confirm the feasibility of completion, reflect the success of the translation and adaptation process into Swahili, and that CHF patients in Tanzania are willing and able to report on their CHF-specific outcomes. The importance of using PROMS in the clinical care and follow-up of CHF patients cannot be overstated [10], with multiple studies showing the ability of PROMS, specifically the KCCQ, to predict various clinical outcomes including mortality [12, 20, 21]. In a low income country like Tanzania, where conventional laboratory and imaging modalities for CHF are limited [3, 31], the KCCQ can be an important additional tool to risk-stratify CHF patients, identify symptoms impacting lower quality of life and support the shared decision making for customized treatment plans.

We found that CHF patients attending their regular follow-up or new appointments at this clinic had generally very poor to poor scores on the KCCQ-23. These findings are remarkably similar to findings in Uganda (mean overall KCCQ-23 score at baseline visit 21.8 points) [20], reflecting similarities in these two patient populations and the general observation that CHF patients in care in African countries have worse symptoms compared to other regions of the world [21, 31]. In the current study, self-efficacy was the best scored measure (mean score 40.7), with lowest scores in social limitation (mean score 15.3), while the overall quality of life was also poor to fair (mean score 27.1). These findings are also similar to the Ugandan study [20]. The explanation for generally poor health outcomes among African CHF patients is multifactorial as previously documented [20, 21], but may also be reflecting a delay in accessing care as well as lack of effective therapies in this population [3, 24, 31]. Further work to understand the causes of these scores, including use on a larger sample and correlation with clinical studies such as echocardiography and adherence to medications is planned.

Studies from HICs have highlighted the challenges of integrating PROMS into clinical care, including within cardiology. For example, Wohlfarht found that cardiology providers from the United States identified three such barriers as: the burden of data collection, ambiguity in interpreting and presenting scores, and defining the tools’ utility and value [32]. There is however growing work in these settings for how to effectively implement the measurement and importantly the use of these tools in patient care [33], although there are still limited results from lower-resourced settings. Our group is working to expand our research to include testing strategies to incorporate KCCQ into clinical care including inclusion of the tool into the hospital’s health information management systems and training of providers to increase use of PROMS in the discussions with patients and care plans. Identifying these strategies will be critical to achieve the goals of PROs to empower patients to report symptoms and their impact on quality of life and functioning and inform more people-centered care which is needed for effective care of people with chronic conditions including CHF. The correlation between the Swahili KCCQ-23 and KCCQ-12 is similar to the correlation between the original English KCCQ-23 and KCCQ-12 [8, 9]. The ability to use a shorter tool could streamline the data collection and reduce the burden on patients and providers, potentially increasing its uptake into clinical care. This work is important to achieve the goals of increasing patient-centred care in our LMIC clinic setting with the potential to roll-out the implementation to other CHF clinics in Tanzania and East Africa [34].

Our study had a number of limitations. Our sample was small, and limited our power to identify patient factors that were associated with the level of symptoms. To address this limitation, work to expand the use in the clinic and other settings is planned. Since the current study was conducted in a referral hospital which is likely to have individuals with more advanced disease, it limited the generalizability of the results. Therefore, the tool needs to also be tested in other settings where CHF patients receive care including primary care clinics. Finally, we did not measure acceptability or feasibility of integrating survey administration and result interpretation into routine care, work which is planned in the future.

## Conclusion

We have successfully translated a validated tool for measuring PROMS among people with CHF in patients with CHF in Tanzania. The translated tool was able to be self-administered, with high rates of self-reported heart failure-related morbidity. This work provides opportunities for expanded PROM use in other countries through the availability of the Swahili KCCQ. Important planned next steps include expanding the population with PROs measured to better understand variability based on socio-demographics, and identifying the strategies needed for integration into clinical care and use for broader quality improvement initiatives in order to improve outcomes among Swahili-speaking patients with CHF.

## Electronic supplementary material

Below is the link to the electronic supplementary material.

Additional File 1: Bivariate Analysis of the KCCQ-23 total scores and Patient? Characteristics

Additional File 2: Dodoso la Ugonjwa wa Misuli ya moyo la Kansas City (KCCQ)

## Data Availability

The datasets generated and/or analyzed during the current study are not publicly available due to privacy and ethical concerns but are available from the corresponding author, Dr. Pilly Chillo on reasonable request.

## List of Abbreviations

CHF: Congestive Heart Failure
HICs: High Income Countries
HIV: Human Immunodeficiency Virus
JKCI: Jakaya Kikwete Cardiac Institute
KCCQ: Kansas City Cardiomyopathy Questionnaire
LMICs: Low and Middle Income Countries
NYHA: New York Heart Association
OECD: Organization for Economic Cooperation and Development
PROMS: Patient-Reported Outcome Measures
PROs: Patient-reported Outcomes
SSA: Sub-Saharan Africa
WHO: World Health Organization
