# Supplementary material for: Adapting and usability testing of the Kansas city cardiomyopathy questionnaire (KCCQ) in a heart failure clinic in Tanzania: the Swahili KCCQ
Source: BMC Cardiovasc Disord. 2023 May 6;23:242. doi: 10.1186/s12872-023-03265-0 (PMC10163850; doi:10.1186/s12872-023-03265-0)
Supplement: Supplementary file 1 — Additional File 1: Bivariate Analysis of the KCCQ-23 total scores and Patient? Characteristics [file 12872_2023_3265_MOESM1_ESM.docx]

**Appendix 2:**

Bivariate Analysis of the KCCQ-23 total scores and Patient’ Characteristics

|  | **KCCQ-23 total score**  **Median** | **p–value** |
| --- | --- | --- |
| **Sex** |  |  |
| Male | 18.75 | 0.31 |
| Female | 12.90 |  |
| **Comorbidity (HTN, DM or kidney disease)** |  |  |
| Yes | 18.23 | 0.54 |
| No | 15.50 |  |
| **NYHA** |  |  |
| Class I & II | 15.00 | 0.55 |
| Class III & IV | 17.71 |  |
| **Education** |  |  |
| Completed Primary school or lower | 16.15 | 0.56 |
| Any secondary or higher | 18.49 |  |
| **Age** |  |  |
| ≤49 | 14.58 | 0.71 |
| 50+ | 18.23 |  |
| **Visit type** |  |  |
| First visit | 17.32 | 0.89 |
| Not first visit | 17.71 |  |
