# Supplementary material for: Adapting and usability testing of the Kansas city cardiomyopathy questionnaire (KCCQ) in a heart failure clinic in Tanzania: the Swahili KCCQ
Source: BMC Cardiovasc Disord. 2023 May 6;23:242. doi: 10.1186/s12872-023-03265-0 (PMC10163850; doi:10.1186/s12872-023-03265-0)
Supplement: Supplementary file 2 — Additional File 2: Dodoso la Ugonjwa wa Misuli ya moyo la Kansas City (KCCQ) [file 12872_2023_3265_MOESM2_ESM.pdf]

Dodoso la Ugonjwa wa Misuli ya moyo la Kansas City (KCCQ)

Maswali yafuatayo yanahusu **ugonjwa** wako **wa moyo** na jinsi unavyoweza kuathiri maisha yako. Tafadhali soma na jibu maswali yafuatayo. Hakuna majibu sahihi au yasiyo sahihi. Tafadhali weka alama jibu unaloona ni sahihi kwako.

1. **Ugonjwa wa moyo** huaathiri watu tofauti kwa namna tofauti. Wengine huhisi upungufu wa pumzi na wengine huhisi uchovu. Tafadhali onyesha ni kiasi gani unaathiriwa na **ugonjwa wa moyo** (kupungukiwa pumzi au uchovu) katika uwezo wako kufanya shughuli zifuatazo katika wiki 2 zilizopita.

Weka alama **X** kwenye kisanduku kimoja katika kila mstari

**Shughuli**	**Unanizuia Mno**	**Unanizuia Kidogo**	**Unanizuia Kiasi**	**Unanizuia Kidogo Sana**	**Haunizuii Kabisa**	**Nazuiwa na sababu nyingine au sikufanya shughuli**
Kujivisha	❑	❑	❑	❑	❑	❑
Kuoga	❑	❑	❑	❑	❑	❑
Kutembea mita 100 eneo la tambarare	❑	❑	❑	❑	❑	❑
Kufanya kazi za mazingira, kazi za nyumbani au kubeba mahitaji ya nyumbani	❑	❑	❑	❑	❑	❑
Kupanda ngazi bila kupumzika	❑	❑	❑	❑	❑	❑
Kutembea kwa haraka au mchaka-mchaka (kama kukimbilia basi)	❑	❑	❑	❑	❑	❑

2. Ikilinganishwa na wiki 2 zilizopita, dalili zako za **ugonjwa wa moyo** (kupungukiwa pumzi, uchovu, au kuvimba vifundo vya miguu) zimebadilika?

Dalili zangu za **ugonjwa wa moyo** zimekuwa…

Mbaya sana	Mbaya kidogo	Hazijabadilika	Nzuri kidogo	Nzuri sana	Sikuwa na dalili ndani ya wiki 2 zilizopita
❑	❑	❑	❑	❑	❑

Dodoso la Ugonjwa wa Misuli ya moyo la Kansas City (KCCQ). Haki miliki ©1992-2006 John Spertus, MD, MPH

3. Katika wiki 2 zilizopita, mara ngapi **umevimba** miguu, vifundo vya miguu au miguu yote ulipoamka asubuhi?

Kila asubuhi	Mara 3 au zaidi kwa wiki, lakini si kila siku	Mara 1-2 kwa wiki	Chini ya mara moja kwa wiki	Kamwe katika wiki 2 zilizopita
❑	❑	❑	❑	❑

4. Katika wiki 2 zilizopita, kwa kiasi gani **uvimbe** wa miguu, vifundo vya miguu au miguuni pako umekusumbua?

Umekuwa…

Ukinisumbua **Mno**	Ukinisumbua **kidogo**	Ukinisumbua **kiasi**	Ukinisumbua **kidogo sana**	Haukunisumbua **kabisa**	Sikuwa na **uvimbe**
❑	❑	❑	❑	❑	❑

5. Katika wiki 2 zilizopita, kwa wastani, ni mara ngapi **uchovu** umezuia uwezo wako kufanya unachotaka?

Wakati wote	Mara kadhaa kwa siku	Angalau mara moja kwa siku	Mara 3 au zaidi kwa wiki lakini si kila siku	Mara 1-2 kwa wiki	Chini ya mara moja kwa wiki	Kamwe katika wiki 2 zilizopita
❑	❑	❑	❑	❑	❑	❑

6. Katika wiki 2 zilizopita, ni kwa kiasi gani **uchovu** wako umekusumbua?

Umekuwa...

Ukinisumbua **Mno**	Ukinisumbua **kidogo**	Ukinisumbua **kiasi**	Ukinisumbua **kidogo sana**	Haukunisumbua **kabisa**	Sikuwa na **uchovu**
❑	❑	❑	❑	❑	❑

7. Katika wiki 2 zilizopita, kwa wastani, ni mara ngapi **upungufu wa pumzi** umezuia uwezo wako kufanya kile ulichotaka?

Wakati wote	Mara kadhaa kwa siku	Angalau mara moja kwa siku	Mara 3 au zaidi kwa wiki lakini si kila siku	Mara 1-2 kwa wiki	Chini ya mara moja kwa wiki	Kamwe katika wiki 2 zilizopita
❑	❑	❑	❑	❑	❑	❑

Dodoso la Ugonjwa wa Misuli ya moyo la Kansas City (KCCQ). Haki miliki ©1992-2006 John Spertus, MD, MPH

8. Katika wiki 2 zilizopita, ni kwa kiasi gani **upungufu wa pumzi** umekusumbua?

Umekuwa...

Ukinisumbua **Mno**	Ukinisumbua **kidogo**	Ukinisumbua **kiasi**	Ukinisumbua **kidogo sana**	Haukunisumbua **kabisa**	Sikuwa na **upungufu wa pumzi**
❑	❑	❑	❑	❑	❑

9. Katika wiki 2 zilizopita, kwa wastani, ni mara ngapi umelazimika kulala ukiwa umeketi kwenye kiti au na mito angalau mitatu kukunyanyua juu sababu ya **kupungukiwa pumzi**?

Kila usiku	Mara 3 au zaidi kwa wiki, lakini si kila siku	Mara 1-2 kwa wiki	Chini ya mara moja kwa wiki	Kamwe katika wiki 2 zilizopita
❑	❑	❑	❑	❑

10. Dalili za **ugonjwa wa moyo** zinaweza kuwa mbaya zaidi kwa sababu nyingi. Una uhakika gani kwamba unajua cha kufanya, au nani wa kumuita ikiwa **ugonjwa** wako **wa moyo** ukizidi?

Sina hakika **kabisa**	Sina hakika **sana**	Nina hakika **kiasi fulani**	Nina hakika **sana**	Nina hakika **kabisa**
❑	❑	❑	❑	❑

11. Kwa kiasi gani unaelewa mambo unayoweza kufanya kuzuia dalili za **ugonjwa wako wa moyo** kuwa mbaya zaidi? (Kwa mfano, kujipima uzito mwenyewe, kula vyakula vyenye chumvi kidogo n.k)

Sielewi kabisa	Sielewi vizuri sana	Naelewa kiasi fulani	Naelewa sana	Naelewa kabisa
❑	❑	❑	❑	❑

12. Katika wiki 2 zilizopita, kwa kiasi gani **ugonjwa wako wa moyo** umekuzuia kufurahia maisha?

Umezuia **mno** furaha yangu ya maisha	Umezuia furaha yangu ya maisha **kidogo**	Kwa **kiasi** umenizuia kufurahia maisha	**Kidogo sana** umenizuia kufurahia maisha	**Haujazuia** furaha yangu ya maisha hata kidogo
❑	❑	❑	❑	❑

13. Kama utaishi maisha yako yote na **ugonjwa** wako **wa moyo** kwa namna ilivyo sasa, utajisikiaje kuhusu hili?

Siridhiki kabisa	Sijaridhishwa hasa	Naridhika kiasi fulani	Naridhika sana	Naridhika kabisa
❑	❑	❑	❑	❑

Dodoso la Ugonjwa wa Misuli ya moyo la Kansas City (KCCQ). Haki miliki ©1992-2006 John Spertus, MD, MPH

14. Katika wiki 2 zilizopta, mara ngapi umejisikia kukata tamaa au kusononeka kwa sababu ya **ugonjwa** wako **wa moyo**?

Nimejisikia hivyo **wakati wote**	Nimejisikia hivyo **muda mwingi**	Nimejisikia hivyo **mara kadhaa**	Kwa **nadra** nimejisikia hivyo	Sijajisikia hivyo **kamwe**
❑	❑	❑	❑	❑

15. Kiasi gani **ugonjwa** wako **wa moyo** unaathiri mfumo wako wa maisha? Tafadhali onyesha ni kiasi gani **ugonjwa** wako **wa moyo** umeweza kukuzuia kushiriki kwenye shughuli zifuatazo katika wiki 2 zilizopita.

Tafadhali weka alama **X** kwenye kisanduku kimoja katika kila mstari.

**Shughuli**	Umenizuia **mno**	Umenizuia **kidogo**	Umenizuia **kiasi**	Umenizuia **kidogo sana**	**Haujanizuia** kabisa	Haihusiki au sikufanya kwa sababu nyingine
Mambo ninayopenda, shughuli za kujiburudisha	❑	❑	❑	❑	❑	❑
Kufanya kazi au majukumu ya nyumbani	❑	❑	❑	❑	❑	❑
Kutembelea familia au marafiki nje ya nyumbani mwako	❑	❑	❑	❑	❑	❑
Mahusiano ya karibu sana na wapendwa	❑	❑	❑	❑	❑	❑

Dodoso la Ugonjwa wa Misuli ya moyo la Kansas City (KCCQ). Haki miliki ©1992-2006 John Spertus, MD, MPH

APPENDIX

THE KANSAS CITY CARDIOMYOPATHY QUESTIONNAIRE:

The following questions refer to your **heart failure** and how it may affect your life. Please read and complete the following questions. There are no right or wrong answers. Please mark the answer that best applies to you.

1. **Heart failure** affects different people in different ways. Some feel shortness of breath while others feel fatigue. Please indicate how much you are limited by **heart failure** (shortness of breath or fatigue) in your ability to do the following activities over the past 2 weeks.

Place an **X** in one box on each line

**Activity**	**Extremely Limited**	**Quite a bit Limited**	**Moderately Limited**	**Slightly Limited**	**Not at all Limited**	**Limited for other reasons or did not do the activity**
Dressing yourself	❑	❑	❑	❑	❑	❑
Showering/Bathing	❑	❑	❑	❑	❑	❑
Walking 1 block on level ground	❑	❑	❑	❑	❑	❑
Doing yardwork, housework or carrying groceries	❑	❑	❑	❑	❑	❑
Climbing a flight of stairs without stopping	❑	❑	❑	❑	❑	❑
Hurrying or jogging (as if to catch a bus)	❑	❑	❑	❑	❑	❑

2. Compared with 2 weeks ago, have your symptoms of **heart failure** (shortness of breath, fatigue or ankle swelling) changed?

My symptoms of **heart failure** have become . . .

Much worse	Slightly worse	Not changed	Slightly better	Much better	I've had no symptoms over the last 2 weeks
❑	❑	❑	❑	❑	❑

3. Over the past 2 weeks, how many times did you have **swelling** in your feet, ankles or legs when you woke up in the morning?

Every morning	3 or more times a week, but not every day	1–2 times a week	Less than once a week	Never over the past 2 weeks
❑	❑	❑	❑	❑

4. Over the past 2 weeks, how much has **swelling** in your feet, ankles or legs bothered you?

It has been . . .

**Extremely** bothersome	**Quite a bit** bothersome	**Moderately** bothersome	**Slightly** bothersome	**Not at all** bothersome	I've had **no swelling**
❑	❑	❑	❑	❑	❑

5. Over the past 2 weeks, on average, how many times has **fatigue** limited your ability to do what you want?

All of the time	Several times per day	At least once a day	3 or more times per week but not every day	1–2 times per week	Less than once a week	Never over the past 2 weeks
❑	❑	❑	❑	❑	❑	❑

6. Over the past 2 weeks, how much has your **fatigue** bothered you?

It has been . . .

**Extremely** bothersome	**Quite a bit** bothersome	**Moderately** bothersome	**Slightly** bothersome	**Not at all** bothersome	I've had **no fatigue**
❑	❑	❑	❑	❑	❑

7. Over the past 2 weeks, on average, how many times has **shortness of breath** limited your ability to do what you wanted?

All of the time	Several times per day	At least once a day	3 or more times per week but not every day	1–2 times per week	Less than once a week	Never over the past 2 weeks
❑	❑	❑	❑	❑	❑	❑

8. Over the past 2 weeks, how much has your **shortness of breath** bothered you?
It has been . . .

**Extremely** bothersome	**Quite a bit** bothersome	**Moderately** bothersome	**Slightly** bothersome	**Not at all** bothersome	I've had **no shortness of breath**
❑	❑	❑	❑	❑	❑

9. Over the past 2 weeks, on average, how many times have you been forced to sleep sitting up in a chair or with at least 3 pillows to prop you up because of **shortness of breath**?

Every night	3 or more times a week, but not every day	1–2 times a week	Less than once a week	Never over the past 2 weeks
❑	❑	❑	❑	❑

10. **Heart failure** symptoms can worsen for a number of reasons. How sure are you that you know what to do, or whom to call, if your **heart failure** gets worse?

**Not at all** sure	**Not very** sure	**Somewhat** sure	**Mostly** sure	**Completely** sure
❑	❑	❑	❑	❑

11. How well do you understand what things you are able to do to keep your **heart failure** symptoms from getting worse? (for example, weighing yourself, eating a low salt diet, etc.)

Do not understand at all	Do not understand very well	Somewhat understand	Mostly understand	Completely understand
❑	❑	❑	❑	❑

12. Over the past 2 weeks, how much has your **heart failure** limited your enjoyment of life?

It has **extremely** limited my enjoyment of life	It has limited my enjoyment of life **quite a bit**	It has **moderately** limited my enjoyment of life	It has **slightly** limited my enjoyment of life	It has **not limited** my enjoyment of life at all
❑	❑	❑	❑	❑

13. If you had to spend the rest of your life with your **heart failure** the way it is right now, how would you feel about this?

Not at all satisfied	Mostly dissatisfied	Somewhat satisfied	Mostly satisfied	Completely satisfied
❑	❑	❑	❑	❑

14. Over the past 2 weeks, how often have you felt discouraged or down in the dumps because of your **heart failure**?

I felt that way **all of the time**	I felt that way **most of the time**	I **occasionally** felt that way	I **rarely** felt that way	I **never** felt that way
❑	❑	❑	❑	❑

15. How much does your **heart failure** affect your lifestyle? Please indicate how your **heart failure** may have limited your participation in the following activities over the past 2 weeks.

Please place an **X** in one box on each line

**Activity**	**Severely** limited	Limited **quite a bit**	**Moderately** limited	**Slightly** limited	**Did not** limit at all	Does not apply or did not do for other reasons
Hobbies, recreational activities	❑	❑	❑	❑	❑	❑
Working or doing household chores	❑	❑	❑	❑	❑	❑
Visiting family or friends out of your home	❑	❑	❑	❑	❑	❑
Intimate relationships with loved ones	❑	❑	❑	❑	❑	❑
